# Potential Molecular Targets for Narrow-Spectrum Agents to Combat *Mycoplasma pneumoniae* Infection and Disease

**DOI:** 10.3389/fmicb.2016.00205

**Published:** 2016-02-25

**Authors:** Mitchell F. Balish, Steven L. Distelhorst

**Affiliations:** Department of Microbiology, Miami UniversityOxford, OH, USA

**Keywords:** mycoplasma, antibiotics, toxins, metabolism, adherence

## Abstract

As *Mycoplasma pneumoniae* macrolide resistance grows and spreads worldwide, it is becoming more important to develop new drugs to prevent infection or limit disease. Because other mycoplasma species have acquired resistance to other classes of antibiotics, it is reasonable to presume that *M. pneumoniae* can do the same, so switching to commonly used antibiotics like fluoroquinolones will not result in forms of therapy with long-term utility. Moreover, broad-spectrum antibiotics can have serious consequences for the patient, as these drugs may have severe impacts on the natural microbiota of the individual, compromising the health of the patient either short-term or long-term. Therefore, developing narrow-spectrum antibiotics that effectively target only *M. pneumoniae* and no more than a small portion of the microbiota is likely to yield impactful, positive results that can be used perhaps indefinitely to combat *M. pneumoniae*. Development of these agents requires a deep understanding of the basic biology of *M. pneumoniae*, in many areas deeper than what is currently known. In this review, we discuss potential targets for new, narrow-spectrum agents and both the positive and negative aspects of selecting these targets, which include toxic molecules, metabolic pathways, and attachment and motility. By gathering this information together, we anticipate that it will be easier for researchers to evaluate topics of priority for study of *M. pneumoniae*.

## Introduction

The use of antibiotics to treat bacterial infections is predicated on the antibiotics’ ability to inhibit significant cellular processes of the bacteria, but not of the host, while avoiding inactivation by the bacteria. For cell wall-lacking mycoplasmas like *Mycoplasma pneumoniae*, a wide range of antibiotics, excluding those that target synthesis of peptidoglycan and certain metabolic pathways, is potentially useful in fighting infection. In practice, treatment of patients is largely restricted to macrolides, with tetracycline and fluoroquinolones used in some geographical regions or under some conditions (Bébéar et al., 2011; Biondi et al., 2014). Indeed, macrolides like azithromycin have historically been very effective against *M. pneumoniae*.

However, like so many other bacterial pathogens, *M. pneumoniae* has recently experienced a rapid increase in the incidence of resistance to the antibiotics commonly used to treat infections (Principi and Esposito, 2013). At present, there are few reports of resistance of *M. pneumoniae* to antibiotics other than macrolides, and they are restricted to *in vitro* studies (Dégrange et al., 2008), but the rise of resistance of related mycoplasma species to fluoroquinolones and tetracycline (Gerchman et al., 2008; Redelinghuys et al., 2014) strongly suggests that *M. pneumoniae* is capable of developing resistance to other drugs if they become standard, widespread means of treatment. Moreover, for prevention of infection, no successful vaccine against *M. pneumoniae* has been developed. Consequently, the time is coming when alternative agents will have to be employed to prevent *M. pneumoniae* infection and to treat patients who are suffering from disease caused by this organism.

Switching to alternative, currently available antibiotics as a normal course of treatment for *M. pneumoniae* infection could be expected to provide some relief, but it is likely inevitable that the organism will develop resistance. Broad-spectrum antibiotics can bring undesirable side effects, often stemming from large-scale disruption of the host microbiome, that can cause problems whose difficulty exceeds those associated with the original infection (Modi et al., 2014). Furthermore, the selective pressure that broad-spectrum antibiotics apply to so many organisms causes resistance to develop fast and spread rapidly among different bacteria. Therefore, it is beneficial to use knowledge of the biochemistry and physiology of *M. pneumoniae* to design and develop narrow-spectrum therapeutic agents that target *M. pneumoniae* as specifically as possible. Whereas some such agents might be used to eradicate the organism, others might target *M. pneumoniae* processes that, although not essential for the life of the bacterium, exacerbate disease, and by so doing both reduce the symptoms and give the patient’s immune system an advantage in clearing the infection.

The depth of understanding of the biology of *M. pneumoniae* has increased dramatically in recent years, thanks in large part to genomics, systems biology, and cell biology studies of this organism. It has become possible to consider, in a more informed way than ever, which activities of *M. pneumoniae* might provide the best targets for development of new, narrow-spectrum drugs. In this review, we will discuss the biology of *M. pneumoniae* in terms of which metabolic pathways, cellular components, and activities are likely to be suitable for future work in this area.

## Targets

Considerable variation exists in the degrees to which potentially important therapeutic targets are understood. Toxins and toxic metabolites are in some ways the most welcoming for study because they involve a small number of proteins and often effectuate biochemical changes that are readily measurable. More complex metabolic pathways are less well-studied and warrant a greater effort. Cell-level processes like adherence, motility, and division are fairly well-characterized but the molecular basis for each of these activities is generally poorly established. In addition to the question of how well any putative inhibitor of a given activity would interfere with the life processes of an *M. pneumoniae* cell, another important consideration is how narrowly a drug would target *M. pneumoniae*. If the target is something that is found, for example, only in mycoplasmas, one would anticipate the ideal outcome of a very narrow-spectrum drug that does not interfere with other components of the host microbiota. Alternatively, if the target is broadly present in bacteria, the narrowness of the drug’s action would depend on whether it can exploit structural differences in the *M. pneumoniae* version of the target. If the structure of such a target is highly conserved, then it may be difficult to develop a therapeutic agent that does not cause disruption to the host by damaging the host microbiota. However, a widely distributed target that exhibits considerable difference in sequence may be more suitable. In **Table 1**, we address these issues for each of the targets discussed in this review.

**Table 1:** Evaluation of potential *Mycoplasma pneumoniae* targets.

Category	Gene number	Protein name	Proposed function	Is the role or function established in *M. pneumoniae*?	Narrowness of phylogenetic distribution
Toxin	MPN372	CARDS toxin	ADP-ribosylating toxin	Yes	High
Toxic metabolites	MPN051	G3P oxidase	Hydrogen peroxide production	Yes	Medium–high
Toxic metabolites	MPN487	Cysteine desulfurase/desulfhydrase	Hydrogen sulfide production	Moderately	Medium–high
Transport	MPN415-417		Thiamine transport	No	Medium
Transport	MPN043	Glycerol facilitator	Glycerol transport	Yes	Medium–high
Transport	MPN133		Glycerol transport (accessory)	Moderately	High
Transport	MPN284		Glycerol transport (accessory)	Moderately	High
Transport	MPN421	Glycerophosphocholine transporter	Glycerophosphocholine transport	Yes	High
Transport	MPN076		Glycerophosphocholine transport (accessory)	Moderately	High
Transport	MPN077		Glycerophosphocholine transport (accessory)	Moderately	High
Anabolism	MPN336	Pantothenate kinase/nicotinate-nucleotide adenylyltransferase	CoA synthesis	No	Low–medium
Anabolism	MPN382	Dephospho-CoA kinase	CoA synthesis	No	Low
Anabolism	MPN298	Acyl carrier protein synthase	Lipid synthesis	Yes	Low
Anabolism	MPN406	Acyl carrier protein	Lipid synthesis	Yes	Low
Anabolism	MPN420	Glycerophosphocholine phosphodiesterase	Lipid synthesis and hydrogen peroxide production	Yes	Medium–high
Anabolism	MPN350	G3P acyltransferase	Lipid synthesis	No	Low
Anabolism	MPN299	1-acyl-G3P acyltransferase	Lipid synthesis	No	Low
Anabolism	MPN483	Glycosyltransferase	Polysaccharide synthesis	Moderately	High
Anabolism	MPN028	Glycosyltransferase	Polysaccharide synthesis	No	High
Anabolism	MPN075	Glycosyltransferase	Polysaccharide synthesis	No	High
Anabolism	MPN073	PRPP synthetase	Nucleotide synthesis	No	Low
Anabolism	MPN066		Nucleotide synthesis	No	Medium–high
Anabolism	MPN256	CTP synthetase	Nucleotide salvage	No	High
AO	MPN141	P1 adhesin	Adherence and motility	Moderately	High
AO	MPN142	Protein B/protein C	Adherence and motility	Moderately	High
AO	MPN626	Alternative sigma factor	Recombination of adherence and motility genes	No	High
AO	MPN453	P30 adhesin	Adherence and motility	Moderately	High
AO	MPN446	HMW1	AO core	Moderately	High
AO	MPN310	HMW2	AO core	Moderately	High
AO	MPN309	P65	AO core	No	High
AO	MPN311	P41	AO core	No	High
Cell division	MPN317	FtsZ	Cytokinesis	Moderately	Medium

### Toxins and Toxic Metabolites

Host cell damage by *M. pneumoniae* is established to occur by several routes. One, which is beyond the scope of this review, is immunopathology, wherein the organism attracts the cells of the host’s immune system, causing inflammation and host cell damage. Another is the ADP-ribosylating community-acquired respiratory distress syndrome (CARDS) toxin, which, though only relatively recently identified, has come to be considered a major source of cell and tissue damage responsible for a substantial portion of the symptoms of *M. pneumoniae* infection. Damage to host cells from hydrogen peroxide and hydrogen sulfide is also potentially significant.

#### CARDS Toxin

One very promising candidate and target for therapeutic design for the treatment of *M. pneumoniae* infections is CARDS toxin, encoded by MPN372. This 68-kDa protein was initially identified, because of its ability to bind with high affinity to surfactant protein A, a prominent component of pulmonary surfactant (Kannan et al., 2005), but was subsequently characterized as an ADP-ribosylating toxin (Kannan and Baseman, 2006). Incubation of recombinantly produced CARDS toxin with tissue culture cells results in a major increase in ADP-ribosylation of host proteins (Kannan and Baseman, 2006). Upon entry into host cells the toxin activates the NLRP3 inflammasome via ADP-ribosylation, a mechanism likely responsible for the robust inflammation and pathology associated with *M. pneumoniae* infections (Bose et al., 2014). CARDS toxin induces extensive vacuolation in tissue culture cells, tracheal organ cultures, and model host animals in a dose-dependent manner and causes cytopathic effects and inflammatory responses similar to the histopathology and immunopathology seen during *M. pneumoniae* infections both *ex vivo* and *in vitro* (Kannan and Baseman, 2006; Hardy et al., 2009). CARDS toxin-induced vacuoles are derived from late endosomes enriched in Rab9, a host cell GTPase involved in membrane trafficking (Johnson et al., 2011). The cellular damage that results from recombinant CARDS toxin suggests that it is a major virulence factor and likely plays a large role in the pathogenesis of *M. pneumoniae*. Patients with confirmed *M. pneumoniae* infections experience high antibody titers to CARDS toxin, likely due to the localization of a subset of the toxin to the *M. pneumoniae* membrane (Kannan and Baseman, 2006; Kannan et al., 2010; Johnson et al., 2011), suggesting the potential utility of CARDS toxin not only as a target for development of agents that inhibit its activity, but also as a vaccine component.

Community-acquired respiratory distress syndrome toxin has a modular structure, with different regions providing distinct functionality. The X-ray crystal structure of CARDS toxin reveals that the protein is composed of three domains folded in the shape of a triangle (Becker et al., 2015). Domain 1 houses the N-terminal ADP-ribosyltransferase activity; the sequence of the N-terminal region of CARDS toxin shares 27% identity with the pertussis toxin S1 subunit of *Bordetella pertussis*, which is an ADP-ribosyltransferase (Kannan et al., 2005). Domains 2 and 3 form a C-terminal tandem β-trefoil (Becker et al., 2015). The C-terminal domain, whose amino acid sequence does not resemble those of other proteins, is solely responsible for binding and internalization as well as vacuolating activity (Kannan et al., 2014). Deletion of 41 amino acids from the C-terminus of CARDS toxin completely abolished binding and internalization of the protein, indicating the involvement of this region in host-cell receptor binding (Kannan et al., 2014). Thus, the data suggest that domain 3 specifically mediates CARDS toxin binding and entry to host cells (Becker et al., 2015). In addition to binding surfactant protein A, CARDS toxin also associates with the host membrane protein annexin A2, colocalizing with annexin A2 prior to internalization and remaining associated with it after internalization (Somarajan et al., 2014). The importance of this interaction was demonstrated by diminution of binding and entry of CARDS toxin into A549 cells after pretreatment of the cells with anti-annexin A2 antibodies or annexin A2-specific siRNA (Somarajan et al., 2014). Uptake occurs via a clathrin-mediated endocytic pathway in multiple mammalian cell lines (Krishnan et al., 2013; Somarajan et al., 2014). Thus, CARDS toxin, though a single molecule, has multiple distinct functionalities that could be targets for intervention.

Although CARDS toxin is not necessary for colonization or infection its production and quantity show a clear and direct relation to disease severity as indicated by the difference in pathology of different clinical strains expressing varying levels of this toxin (Techasaensiri et al., 2010). Therefore, inhibition of production, cell entry, or activity of CARDS toxin could provide a reprieve to infected patients to allow for immune clearance and significantly less cellular damage.

#### Toxic Metabolites

Glycerol-3-phosphate (G3P) can be used either for synthesis of lipids (see section “Metabolism of G3P”) or conversion to dihydroxyacetone phosphate (DHAP), which enters the glycolytic pathway. The conversion of G3P to DHAP is significant because the enzyme that catalyzes that reaction, G3P oxidase (GlpO, encoded by MPN051), simultaneously reduces molecular oxygen to hydrogen peroxide (Hames et al., 2009; Maenpuen et al., 2015), which is cytotoxic and suggested to be important for virulence of *M. pneumoniae*. *M. pneumoniae* GlpO also uses the glycolytic intermediate glyceraldehyde-3-phosphate as a substrate with a low turnover rate (Maenpuen et al., 2015), explaining the evolution of hydrogen peroxide by *M. pneumoniae* in the absence of glycerol (Hames et al., 2009). Study of recombinantly produced *M. pneumoniae* GlpO revealed significant differences in the active site from the nominally similar mitochondrial G3P dehydrogenase (Elkhal et al., 2015), paving the way toward the use of GlpO as a therapeutic target.

Although hydrogen peroxide is cytotoxic and cell lysis *in vitro* has been attributed to this molecule, hydrogen sulfide has also been implicated in hemolysis by *M. pneumoniae*, raising the possibility that it too is a virulence factor. Hydrogen sulfide is produced from cysteine by HapE, a novel cysteine desulfurase and cysteine desulfhydrase encoded by MPN487 (Grosshennig et al., 2016). Because an enzyme with both these activities has not been described in other organisms, HapE might be a good target for development of narrow-spectrum agents. Although it is not essential *in vitro*, it might nonetheless play important roles in virulence. Further work should be done to establish the importance of HapE and hydrogen sulfide in *M. pneumoniae* pathogenesis.

### Metabolism and Metabolites

Using metabolic pathway inhibitors against *M. pneumoniae* relies on identifying metabolic pathways that are both active and important, if not essential, to the organism, and distinct enough from host metabolic pathways to limit toxicity to the host. However, approaches that work in other bacteria often fail with regard to mycoplasmas. For example, *M. pneumoniae* does not synthesize folate and is therefore insensitive to sulfonamides, which target enzymes involved in its synthesis (McCormack, 1993). The reduced biosynthetic capabilities of *M. pneumoniae* and other mycoplasmas (Himmelreich et al., 1996), coincident with their evolutionarily reduced genomes, make identification of suitable pathways challenging. In short, *M. pneumoniae* is already an expert at acquiring, rather than synthesizing, metabolites, making anabolic targets few.

Transport of essential compounds, including cofactors and building blocks, could provide a reasonable set of targets for the development of therapeutic agents that inhibit *M. pneumoniae*. Many putative transporter genes have been identified, but they are largely orphan transporters whose substrates are unknown. For example, the *M. genitalium* homolog of MPN415 encodes a thiamine-binding lipoprotein, and the remaining genes in its operon, MPN416 and MPN417, encode an ABC transporter, suggesting that this transporter serves to import thiamine (Sippel et al., 2011). If so, then given that *M. pneumoniae* cannot synthesize cofactors like thiamine, this transporter could be an excellent target for interfering with *M. pneumoniae* growth, but it must first be experimentally established that thiamine import is the role of this transporter. Because of their hydrophobicity, complexity, and often their essential nature, transporters like that encoded by MPN416 and MPN417 are difficult to study, but understanding the molecular basis for the transport of essential molecules, including cofactors, amino acids, sugars, and nucleic acid precursors, should be a high priority.

The metabolic pathways of *M. pneumoniae* that seem most likely to yield productive narrow-spectrum agents are the synthesis of phospholipids and glycolipids, with G3P at a significant crossroads between membrane biochemistry and hydrogen peroxide synthesis. The poorly understood role of biofilms and extracellular polysaccharide is of considerable potential in this regard as well. Nucleotide salvage pathways, carotenoid synthesis, and catabolic pathways are also worth consideration.

#### Coenzyme A (CoA) Synthesis and Lipid Catabolism

Coenzyme A synthesis is an interesting potential target for inhibition because membrane biogenesis and modification, important processes in which CoA participates, are fundamental for the viability of cells in general. The value of targeting CoA biosynthesis for antibacterial effects is illustrated by the study of pantothenamides as inhibitors of pantothenate kinase (Strauss and Begley, 2002), which catalyzes the rate-limiting step in CoA synthesis in many bacteria, and the use of pyrazinamide, an anti-tuberculosis agent, which might target CoA metabolism (Zhang et al., 2014). Synthesis of CoA by *M. pneumoniae* from externally provided pantetheine, which would have to be imported through an unknown mechanism, could occur in three steps, beginning with pantothenate kinase (Yus et al., 2009); however, pantothenate kinase activity has not been demonstrated at the biochemical level in *M. pneumoniae* and no gene is confidently annotated as such. Although MPN336, annotated as a nicotinate-nucleotide adenylyltransferase, was proposed as a potential pantetheine phosphate adenylyltransferase, catalyzing synthesis of dephospho-CoA (Yus et al., 2009), global transposon mutagenesis of *M. pneumoniae* clearly revealed the dispensability of this enzyme *in vitro* (Hutchison et al., 1999). On the other hand, there is no evidence that MPN382, which is annotated as dephospho-CoA kinase (CoaE), catalyzing the putative final step in CoA synthesis (Yus et al., 2009), is dispensable (Hutchison et al., 1999). Although this information highlights the potential for development of a CoaE inhibitor, human tissue also uses CoaE, making it important to screen any potential *M. pneumoniae* CoaE inhibitor for low inhibitory activity of its human counterpart. Furthermore, CoaE is used by many bacteria, so a therapeutic agent targeting this molecule might have a broad spectrum of activity, which might not be desirable. In any event, CoA metabolism in *M. pneumoniae* is an insufficiently investigated area that might be of considerable practical value.

Mycoplasmas can acquire fatty acids from host cells, and it is possible that they use them for synthesis of phospholipids and glycolipids (Yus et al., 2009). *M. pneumoniae* has homologs of two proteins that are likely involved in the earliest and most generalized stages of this process, acyl carrier protein synthase, AcpS, encoded by MPN298, and acyl carrier protein, AcpP, encoded by MPN406. These enzymes have both been biochemically characterized with regard to activity and substrate specificity (McAllister et al., 2006). AcpP becomes activated when AcpS catalyzes the transfer of a 4′-phosphopantetheinyl group from CoA to a serine residue on AcpP, and AcpP provides the phosphopantetheine as a cofactor for delivery of acyl groups to a nascent phospholipid or glycolipid. Both their coding genes are suggested to be essential *in vitro* (Hutchison et al., 1999). Recombinantly produced *M. pneumoniae* AcpS catalyzes the pantetheinylation of recombinantly produced AcpP, but with a markedly low affinity for CoA derivatives and, concomitantly, a slow rate of catalysis as compared with AcpS from other bacteria (McAllister et al., 2006). Indeed, it can also transfer a variety of CoA-linked substrate molecules other than phosphopantetheine, although the significance of this broad specificity and slow turnover for *M. pneumoniae* physiology is unclear. These enzymes might be interesting targets for development of novel anti-*M. pneumoniae* agents, but selecting against inhibitors of homologous human lipid synthesis proteins and, for that matter, similar proteins in commensal bacteria, would be an important consideration.

#### Metabolism of G3P

Although some phospholipids are acquired intact from the host or the media, the glycerol backbones of other phospholipids and glycolipids in *M. pneumoniae* are predicted to derive either from glycerol or glycerophospholipids of the host cell membrane (Yus et al., 2009). Exogenous G3P was suggested by gene annotation to be another potential source of glycerol (Himmelreich et al., 1996), in addition to free glycerol, for metabolism, but this was experimentally ruled out (Schmidl et al., 2011). However, both glycerol and glycerophospholipids are converted to G3P in the *M. pneumoniae* cell (Hames et al., 2009; Grosshennig et al., 2013). In addition, metabolism of G3P by another pathway results in production of hydrogen peroxide (see section “Toxic Metabolites”), a virulence factor of *M. pneumoniae* (Hames et al., 2009), so if the bacteria could survive therapeutic agents targeting early stages of glycerol and glycerophospholipid metabolism, they would nonetheless be impaired in their virulence, giving the host a better chance of success against the pathogen.

It is unknown whether during an infection *M. pneumoniae* relies on free glycerol, which is not abundant in the normal environment of the organism, in contrast to glycerophospholipids, which are; consequently, whether metabolism of free glycerol is physiologically significant enough to constitute a reasonable target for new drugs is unclear, making this question a priority for study. *M. pneumoniae* can take up free glycerol, as well as water, through the glycerol facilitator, GlpF, encoded by MPN043, which is essential (Hutchison et al., 1999). Growth on glycerol is negatively affected in mutants of lipoproteins encoded by MPN133 and MPN284, suggesting ancillary roles in glycerol transport for these molecules (Grosshennig et al., 2013), but these proteins are non-essential (Hutchison et al., 1999). Subsequent conversion of imported glycerol to G3P is carried out in *M. pneumoniae* by glycerol kinase, encoded by the essential gene MPN050, at the expense of a mole of ATP per mole of glycerol, making it another potential target (Hames et al., 2009), but one with a human homolog. However, even among mycoplasmas, GlpF has low sequence homology (Pritchard et al., 2014), suggesting that inhibitors specific to mycoplasmas or specifically to *M. pneumoniae* could be developed. Therefore, of the proteins involved in metabolism of free glycerol, the transporter GlpF is likely the most suitable as a novel target for interfering with glycerol uptake. Interestingly, we are unaware of homologs of GlpF having been described as targets for antibiotics.

Glycerol-3-phosphate can also be derived from breakdown of host cell membrane lipids. Although no lipases have been unambiguously identified in *M. pneumoniae*, lipase activity from other bacteria or endogenous activity of the host could provide glycerophosphocholine (GPC). GlpU, encoded by MPN421, is essential for uptake of GPC, presumably acting as a transporter (Grosshennig et al., 2013), and GlpQ, encoded by MPN420, catalyzes removal of the choline from GPC, yielding G3P (Schmidl et al., 2011). Mutants in either of these two genes exhibit greatly reduced cytotoxicity *ex vivo*, and *in vivo* one might anticipate that interference with the function of either of the two proteins by some therapeutic agent would cause *M. pneumoniae* to rely principally on free glycerol for phospholipid, glycolipid, and the vast majority of hydrogen peroxide synthesis, causing considerable impairment in both growth and virulence. The MPN076 and MPN077 genes are also involved in use of GPC, likely in terms of transport (Grosshennig et al., 2013), but their roles are unclear. A homolog of GlpQ encoded by MPN566 does not function in GPC hydrolysis, and its activity is unknown (Schmidl et al., 2011).

#### Membrane Lipids and Glycomoieties

The most reasonable suggested pathway for the synthesis of any phospholipids and glycolipids that are not derived directly from the host begins with G3P (Yus et al., 2009). If G3P is to be used for membrane lipid synthesis, it must receive two acyl groups via the activities of two successively acting acyltransferases. It is proposed that the first is PlsY (MPN350) and the second is PlsC (MPN299; Yus et al., 2009), but the *M. pneumoniae* enzymes have never been characterized, which is an important step toward establishing the physiological relevance of this metabolic pathway and therefore whether these enzymes, as well as any downstream of them, should be considered targets for new antibiotics. Substrate mimics that inhibit *Streptococcus pneumoniae* PlsY have been studied (Grimes et al., 2008), but because these enzymes are so widely distributed, suggesting that it may be difficult to develop narrow-spectrum agents. Uptake of cholesterol is also essential for *M. pneumoniae* (Johnson and Somerson, 1980), and is unlikely to be related to processes that occur in commensal bacteria, making it an excellent target, but there are no data describing the molecular mechanism by which this is accomplished.

Glycomoieties for use in glycolipids, protein glycosylation, or as extracellular polysaccharide are almost certainly the result of poorly defined anabolic pathways in *M. pneumoniae*. Knowledge of the diversity and function of *M. pneumoniae* polysaccharides, both free and covalently linked to other biomolecules, is limited, but the available information suggests important roles for these moieties in several *M. pneumoniae* biological processes, including glycolipid synthesis, modification of proteins, and biofilm properties (Yus et al., 2009; Simmons et al., 2013). Glycolipid biosynthesis by *M. pneumoniae* using exogenously supplied palmitic acid, ceramide, glucose, UDP-glucose, and phosphate has been demonstrated experimentally (Klement et al., 2007). Undoubtedly essential for the synthesis of these polysaccharides are three glycosyltransferases, encoded by MPN028, MPN075, and MPN483, although the activities of the first two are uncharacterized. Strains in which these genes were definitively knocked out were not isolated in a global transposon mutagenesis screen, suggesting that they are essential for *M. pneumoniae* (Hutchison et al., 1999). MPN483 encodes a promiscuous glycosyltransferase that can catalyze the processive synthesis of a variety of polysaccharides from several substrates for use as glycomoieties. When produced recombinantly in *E. coli* it can use UDP-galactose and UDP-glucose as substrates for addition to diacylglycerol, ceramide, and mono-, di-, and trisaccharide derivatives thereof (Klement et al., 2007). The specific identities and significances of its physiological product are unknown.

Significantly, *M. pneumoniae* biofilms grown *in vitro* contain considerable amounts of a polymer of unknown structure enriched in galactose and *N*-acetylglucosamine (Simmons et al., 2013). Although the roles of biofilms in *M. pneumoniae* infection have not been established yet, biofilms of other organisms contribute to virulence, resistance to antibiotics, and susceptibility to clearance by immune system processes, making *M. pneumoniae* biofilms an extremely valuable target for study, especially given the recent increase in antibiotic resistance by *M. pneumoniae* and the chronicity of *M. pneumoniae* infection. Extracellular polysaccharides, such as the one described for *M. pneumoniae*, are often essential features for the formation and integrity of these multicellular structures, and destruction or disruption of their synthesis could be significant means by which *M. pneumoniae* is rendered less virulent or at least more susceptible to other drugs. The polysaccharide that was identified in *M. pneumoniae* is particularly interesting because in a strain that makes a biofilm of reduced density, this molecule is detached from the cells that produce it, whereas a strain that makes a heavier biofilm has this polysaccharide attached to the bacteria, implicating this molecule in important aspects of biofilm integrity (Simmons et al., 2013). Beyond its composition, neither the structure of this polysaccharide nor the enzymes responsible for its synthesis and attachment to the *M. pneumoniae* cell are known. It is unlikely that the glycosyltransferase encoded by MPN483 is involved, given the presence of *N*-acetylglucosamine in it, leaving MPN028 and MPN075 as the most likely candidates for synthesis of the extracellular polysaccharide (Klement et al., 2007). Further work on the characterization and biochemical origin of this molecule is highly warranted.

#### The Question of Carotenoids

Synthesis of carotenoids might be another membrane-associated target, but an insufficient amount of work has been done to establish how important these molecules are and even whether *M. pneumoniae* synthesizes them or acquires them by other means. Carotenoids are membrane-associated pigment molecules with a variety of physiological roles. It is unclear exactly how they might contribute to fitness of *M. pneumoniae*, but a role in protection from photodamage is conceivable. Molecules with Raman spectra consistent with carotenoids were described in multiple *M. pneumoniae* isolates, and a set of genes encoding enzymes involved in their synthesis from the glycolytic intermediates pyruvate and glyceraldehyde-3-phosphate was proposed (Maquelin et al., 2009). However, the specific chemical identities of the final carotenoid products were not described. Maquelin et al. (2009) used analogy with *E. coli* and other organisms that synthesize carotenoids to propose a seven-enzyme pathway for *M. pneumoniae* carotenoid synthesis pathway, but only identified six genes they considered to encode likely participants in this pathway in the *M. pneumoniae* genome. The validity of this pathway has not been addressed experimentally, and some of these genes have annotations that are more consistent with other functions than the ones proposed (Maquelin et al., 2009). If, however, the biological relevance of this pathway could be confirmed and the importance of these molecules for *M. pneumoniae in vivo* could be established, the carotenoid biosynthesis pathway could be a reasonable target for development of drugs. Humans do not synthesize carotenoids, instead acquiring them principally from diet, making at least some enzymes of this pathway stand out as potential targets. For the present time, the biology of carotenoids and their synthesis and/or acquisition by *M. pneumoniae* constitute an interesting topic for further study.

#### Nucleoside/Nucleotide Metabolism

*Mycoplasma pneumoniae* cannot synthesize purines or pyrimidines (Pollack, 2002), which are required for nucleic acid synthesis as well as for anabolic processes involving UDP-and CDP-conjugated carbohydrates. Presumably, like related mycoplasma species, it acquires these molecules from the environment through the use of nucleases and transporters (Li et al., 2010; Masukagami et al., 2013). However, there is potential for interconversion of some of these molecules via nucleotide or nucleoside salvage pathways to ensure that needs are met (Pollack, 2002). It is not clear whether metabolism of these molecules is a suitable target for development of drugs specific to *M. pneumoniae*, because of both a lack of information about *in vivo* flux through various redundant routes and the broad distribution of most of the components of the salvage pathways. Nucleoside analogs that inhibit either bacterial RNA or DNA synthesis or some of the enzymes associated with metabolism of these compounds are widely discussed potential therapeutic agents in the process of development (Niu and Tan, 2015), and their use against mycoplasmas has been proposed as well (Wang et al., 2010, 2014). What remains to be seen is whether effective, narrow-spectrum agents in this category can be developed.

5-phophoribosyl-1-pyrophosphate (PRPP) is a potential source of some nucleotides, providing the sugar for addition to the free bases adenine, guanine, and uracil (Yus et al., 2009). PRPP is a product of the pentose phosphate pathway, which is abbreviated in *M. pneumoniae*, but all the activities that lead to its synthesis from five- and six-carbon sugars are accounted for, although it is unclear what enzyme contributes aldolase activity. Ribulose-5-phosphate (R5P) is the precursor of PRPP, its conversion to PRPP catalyzed by PRPP synthetase, encoded by MPN073 (Yus et al., 2009). R5P itself can derive either from the pentose phosphate pathway or, likely, from ribose-1-phosphate (R1P) yielded by the breakdown of environmental or cytoplasmic RNA. It is likely that MPN066 encodes the enzyme that interconverts R1P and R5P (Yus et al., 2009), but the identity of this protein, which is also annotated as a phosphomannomutase or phosphoglucomutase, has not been experimentally demonstrated. If PRPP can be derived through alternative routes, then a drug targeting either of the pathways might not be useful. Although it is clear that free nucleosides can support the nucleic acid needs for growth of *M. pneumoniae* (Yus et al., 2009), the relative flux through the PRPP pathway as compared with acquisition of nucleosides from nucleic acids has not been established, so even if this pathway could be inhibited, it is unclear what the impact on *M. pneumoniae* would be. The question of how these nucleosides are generated *in vivo* is therefore open, and the uncertainty concerning the activity of MPN066 is an area worth exploring experimentally in connection with this question. Conversion of adenine, guanine, and uracil to their various phosphorylated forms for incorporation into RNA and their interconversion, including into deoxynucleotides for incorporation into DNA, is catalyzed by a series of enzymes that are widely distributed throughout nature and might therefore not be ideal candidates for targets of drugs for narrow-spectrum activity against *M. pneumoniae*.

Pyrimidine metabolism is more likely to provide a good narrow-spectrum target. *M. pneumoniae* appears to lack a CTP synthase enzyme, which would convert UTP to CTP, connecting the PRPP pathway to metabolism of cytosine and thymine nucleotides. MPN256 has been proposed to encode CTP synthase (Yus et al., 2009) and others have proposed the possibility of such an activity (Pachkov et al., 2007), but the evidence is based entirely on bioinformatics approaches and not on biochemical ones. If there is indeed no such activity in *M. pneumoniae*, then the organism must use sources other than PRPP for the generation of cytosine and thymine nucleotides. CDP can be converted to deoxycytidine and thymine nucleotides (Yus et al., 2009), but the ultimate source of CDP must be DNA and RNA or free nucleosides, and not PRPP. Indeed, among pyrimidines, cytosine is sufficient to support growth of *M. pneumoniae* in minimal media (Yus et al., 2009). Drugs based on pyrimidine nucleoside analogs have been suggested as good targets for interfering with growth of *M. pneumoniae* (Wang et al., 2010, 2014), but whether these can be sufficiently specific to avoid killing off commensal microbiota is unknown. Identification and inhibition of the transporters involved in uptake of pyrimidines might also provide useful targets.

#### Catabolic Targets

*Mycoplasma pneumoniae* has genes for generation of ATP through both the arginine dihydrolase pathway and catabolism of sugars. It cannot actually metabolize arginine because of disruption to some of the genes of this pathway in all known strains (Rechnitzer et al., 2013; Xiao et al., 2015). Therefore, arginine catabolism is not a pathway that can be successfully targeted in *M. pneumoniae*. *M. pneumoniae* can utilize a number of sugars as carbon sources for growth *in vitro*, including glucose, mannose, fructose, ribose, ascorbate, glycerol, and possibly mannitol, although there is conflicting information about mannitol (Halbedel et al., 2004; Yus et al., 2009). The relative amount of growth from metabolizing each of these has been established under a defined set of conditions, with glucose and mannose outperforming the others by a considerable margin (Yus et al., 2009). Although it is not clear what the most relevant catabolic pathways *in vivo* are, they would depend upon a combination of substrate availability, affinity of each substrate for its transporter, and the rates of the rate-limiting steps in metabolism of each substrate. Nonetheless, the evolutionary conservation of these metabolic options suggests that they are all useful, and one might anticipate that they come into play at different stages of infection. For example, utilization of ribose by *M. pneumoniae* is likely to increase later in infection after host cell lysis has occurred, making nucleic acids available.

Because of the multiplicity of routes by which carbon sources can be utilized, as well as the common nature of these pathways among bacteria, including commensal ones whose elimination is undesirable, it is likely impractical to consider interfering with the uptake or early stages of metabolism of these compounds. All these metabolic pathways converge on G3P, the entry point into the energy-yielding phase of glycolysis, which is highly conserved and present in the host, and therefore a poor candidate for targeting of drugs.

### Attachment Organelle

**FIGURE 1:**
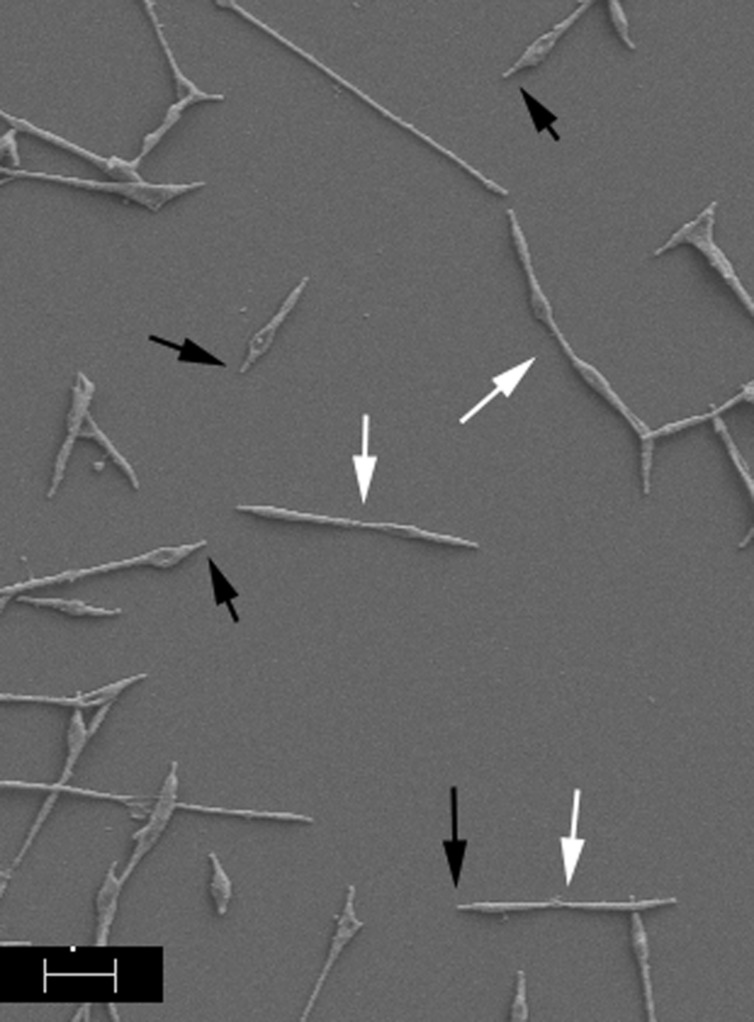
**Scanning electron image of *Mycoplasma pneumoniae* cells**. Cells were prepared according to Hatchel et al. (2006). Black arrows indicate AOs; white arrows indicate dividing cells. Scale bar, 1 μm.

*Mycoplasma pneumoniae* exhibits the properties of cytadherence and gliding motility, and both are required for virulence (Balish, 2014), indicating that drugs designed to interfere with these processes could be of considerable utility in fighting or preventing *M. pneumoniae* disease. A diverse array of molecules across the surface of *M. pneumoniae* cells is involved in host cell adherence but the initial contact and attachment are mediated by a polar structure known as the terminal or attachment organelle (AO), where proteins necessary for *M. pneumoniae* adherence and gliding motility are concentrated (**Figure 1**). Although the identities of at least some of the proteins associated with adherence have been established, the process of gliding motility is less well characterized at the molecular level. The AO contains transmembrane proteins involved in attachment, motility, and perhaps other unknown functions, as well as an interior set of cytoskeletal core proteins (Balish, 2014). Analyses of the proteins of the AO have provided some insight into the spatial and temporal organization of this structure as well as functional characterization of a few of these proteins. Proteins involved in AO synthesis and function are highly specific to a subset of mycoplasma species (Balish, 2014) and therefore provide very narrow targets.

#### Substrate Molecules

The substrates for adherence and gliding motility include sialic acid-containing molecules as well as sulfated glycolipids (Loomes et al., 1984; Krivan et al., 1989; Kasai et al., 2013), suggesting that these molecules could be models for inhibitors of AO function. Although adhesins have been identified, specific interactions between these adhesins and these substrates have never been characterized. This gap in knowledge is due in large part to the absence of protein biochemistry performed on these proteins, illustrating the urgency of performing these studies. The molecular-level means by which the identified adhesins interact with host target molecules will provide targets with great therapeutic potential, but obtaining biochemically active adherence molecules is required for the necessary knowledge. Likewise, how adherence relates to motility at the molecular level is also understood only at a phenomenological level, without deep understanding of the physiological mechanisms. Because adherence and motility are essential for infection, this area of research must be a high priority.

#### Adhesins

One of the most significant proteins of the *M. pneumoniae* AO is the adhesin P1, encoded by MPN141. P1 is distributed across the cell surface but is concentrated at the AO (Baseman et al., 1982). The importance of P1 in host cell attachment is supported by the inhibition of cytadherence when P1 is absent in mutants or blocked by specific antibodies (Krause et al., 1982; Krause and Baseman, 1983). Although P1 is distributed across the cell, the clustering at the AO is necessary for attachment as indicated by mutants that express P1 at wild-type levels but fail to localize the protein to the AO (Baseman et al., 1982; Hahn et al., 1998; Balish et al., 2003).

Protein B (also called P90), a product of the MPN142 gene, colocalizes and copurifies with P1 (Seto and Miyata, 2003; Nakane et al., 2011). Furthermore, MPN142 is cotranscribed with the P1-encoding gene and also required for cytadherence (Krause et al., 1982; Waldo and Krause, 2006). Cleavage of the MPN142 product, which occurs via a process that is unknown, yields proteins B and C (also known as P40); protein C also colocalizes with P1 and is required for cytadherence (Krause et al., 1982; Franzoso et al., 1993; Waldo and Krause, 2006). Although protein C was not copurified with P1 (Nakane et al., 2011), proteins B, C, and P1 can be chemically cross-linked, suggesting that these proteins function together in a complex (Layh-Schmitt and Herrmann, 1994). Therefore it is likely that proteins B and C are involved in contributing to the adhesive property of P1 or that these proteins together function as a single adhesive unit. Both P1 and protein B are immunodominant, suggesting that either one, or perhaps both considered together as a polypeptide adhesin, could be a potential candidate for therapeutic development (Aubert et al., 1992). Additionally, studying the process by which the precursor of proteins B and C is proteolytically cleaved might yield another target.

Of potential significance is the fact that the MPN141 and MPN142 genes are subject to substantial sequence variation across *M. pneumoniae* isolates (Su et al., 1990; Kenri et al., 1999; Spuesens et al., 2011; Xiao et al., 2015). This variation appears to occur as a result of recombination of related variant sequences, located throughout the chromosome, into the expression site (Kenri et al., 1999; Spuesens et al., 2009, 2011). Analogy with a presumably parallel and better characterized system in *M. genitalium* suggests that this diversity represents an antigenic variation scheme (Peterson et al., 1995; Iverson-Cabral et al., 2007; Ma et al., 2007). In *M. genitalium*, homologous recombination is stimulated by the ortholog of *M. pneumoniae* MPN626 acting as a novel sigma factor (Burgos and Totten, 2014; Torres-Puig et al., 2015), suggesting that this protein could also constitute a target for a therapeutic agent that would assist the immune system in clearing infection by blocking antigenic variation from occurring. Characterization of this recombination system in *M. pneumoniae* should therefore be a priority. At the same time, in light of this variation, any drugs that impact the functions of proteins P1, B, or C might select for strains expressing divergent sequences, indicating that any such agents should be tested against a wide variety of strains prior to deployment.

The transmembrane protein P30, encoded by MPN453, is another *M. pneumoniae* adherence protein that is also a good candidate for therapeutic development. Like P1, monoclonal antibodies against P30 inhibit cytadherence (Morrison-Plummer et al., 1986). However, unlike P1, P30 localizes exclusively at the AO (Baseman et al., 1987; Seto et al., 2001). In a mutant that lacks P30 due to a frameshift in MPN453, cells are non-motile, unable to cytadhere, and avirulent (Krause et al., 1982; Romero-Arroyo et al., 1999). Furthermore, a revertant strain, II-3R, in which a second frameshift mutation restores all but 17 amino acids, shows a near-wild-type level of hemadsorption but is almost completely non-motile (Hasselbring et al., 2005), illustrating that P30 has a specific role in gliding motility that is distinct from its role in cytadherence.

The primary structure of P30 is divisible into multiple regions. Its N-terminus, after removal of a long, atypical signal sequence, appears to be in the cytoplasm, and the C-terminus is accessible to carboxypeptidases and therefore located on the cell surface (Dallo et al., 1996; Chang et al., 2011). Mutants with progressive truncation of the P30 C-terminus exhibit decreasing levels of P30 in these mutants as well as drastically decreased gliding motility and cytadherence (Chang et al., 2011). The surface-exposed portion of P30 has an unusually high proline content, with that amino acid constituting 51 of the 125 amino acid residues in this region, and the majority of these prolines are organized into at least 13 sets of 6-amino-acid varying repeats (Dallo et al., 1990). The resulting decrease in the steady-state levels of P30 that lack a number of these repeats suggests that these prolines play a role in stabilizing P30, perhaps by enabling certain interactions or through structural integrity. Truncations in the cytoplasmic N-terminus of P30 remained stable but were unable to restore hemadsorption or gliding motility, suggesting that this region is necessary for proper function while also providing evidence that the C-terminal region has a role in P30 stability not shared with other regions of the protein (Chang et al., 2011). These results indicate the importance of both the internal and external portions of this protein for attachment and gliding motility, which may serve as an important feature in the design of therapeutic agents targeting this protein. Although an effort to create a P30 mutant strain of *M. pneumoniae* as a vaccine strain in an animal model was unsuccessful and in fact resulted in disease exacerbation when mice were infected with a virulent strain of *M. pneumoniae* (Szczepanek et al., 2012), the potential for using P30 in a subunit vaccine or of development of an agent capable of interfering with P30 function remains an option.

#### Internal AO Components

The specific mechanisms by which P1 and P30 are concentrated at the AO is unknown but it appears to depend heavily upon the cytoskeletal core of the AO. The core is composed of a set of cytoskeletal proteins necessary for development, structure, and proper localization of *M. pneumoniae* adhesins. These proteins, including HMW1, HMW2, HMW3, P28, P41, P200, P65, and TopJ, form a complex ordered network of interdependent interactions (Balish, 2014; Nakane et al., 2015). These proteins are necessary for proper development and function of the AO and are organized spatially (Nakane et al., 2015) and assemble in a temporal sequence (Krause and Balish, 2004). Two structural proteins that are required early in the AO assembly process, HMW1 (MPN446) and HMW2 (MPN310), have a special role in localization of P1 to the AO (Balish et al., 2003). When AO protein P65 (MPN309) is disrupted, structures containing P30 detach from *M. pneumoniae* cells, illustrating the importance of P65 for P30 localization and function (Hasselbring et al., 2012). Interestingly, loss of protein P41 (MPN311) causes the entire AO to be susceptible to release from cells during motility (Hasselbring and Krause, 2007), demonstrating the breadth of significant structural roles that AO core proteins have. Therefore, any and all AO core proteins are also potential therapeutic targets. Although the density, compactness, and cytoskeletal nature of the core make them potentially difficult for small molecules to reach, further knowledge about how these proteins assemble and interact may ultimately make it possible to design agents that target them before they become incorporated into nascent AO cores, thereby inhibiting formation of the AO.

### Cell Division

Bacterial cell division is best understood in the context of cell wall biosynthesis; the absence of peptidoglycan in *M. pneumoniae* has rendered this process somewhat enigmatic. In model bacteria, the protein FtsZ forms cytoskeletal polymers at the division site, and these polymers, as components of a division machine, the divisome, coordinate rounds of iterative membrane invagination with local cell wall construction (Lutkenhaus et al., 2012). The *M. pneumoniae* genome includes a gene, MPN317, encoding a highly divergent FtsZ, whose expression levels are extremely low, at less than one mRNA per cell (Benders et al., 2005). Because cell wall synthesis is linked to FtsZ function in other bacteria, it is unclear specifically how FtsZ could contribute to efficient cell division in mycoplasmas, given the absence of peptidoglycan. Indeed, a knockout of this gene’s ortholog in *M. genitalium* did not inhibit cell division but appeared to cause the cells to rely entirely on gliding motility to achieve cytokinesis (Lluch-Senar et al., 2010). Thus, the relationship of FtsZ to cell division in *M. pneumoniae* is unclear.

FtsZ has become a popular target for the development of new antibacterial agents (den Blaauwen et al., 2014). The sequence divergence of *M. pneumoniae* FtsZ, reflected in its inability to complement an *E. coli ftsZ* mutant (Osawa and Erickson, 2006), potentially makes this protein suitable as a target in terms of narrowness, since inhibitors might be specific to divergent features of the protein. However, the low expression levels of MPN317, uncertain relationship of the protein to the actual cell division process, and absence of knowledge about its role in virulence of *M. pneumoniae* raise questions about its suitability that are potentially resolvable with further study.

## Conclusion

Despite a reduced genome and a small number of biosynthetic pathways, *M. pneumoniae* provides ample potential targets for development of narrow-spectrum agents to combat disease caused by this unusual bacterium. Some, like CARDS toxin and adhesins, are reasonably well-studied and could provide excellent substrates for inhibition by new drugs. For others, the physiological and biochemical details are lacking, but the gaps in knowledge provide numerous opportunities for research whose ultimate goal is to develop targets for fighting disease.

## Author Contributions

MB and SD analyzed and interpreted data and drafted the manuscript.

## Conflict of Interest Statement

The authors declare that the research was conducted in the absence of any commercial or financial relationships that could be construed as a potential conflict of interest.
